# Technology-based approaches toward a better understanding of neuro-coagulation in brain homeostasis

**DOI:** 10.1007/s00441-021-03560-2

**Published:** 2021-12-01

**Authors:** Ben M. Maoz, Maria Asplund, Nicola Maggio, Andreas Vlachos

**Affiliations:** 1 Department of Biomedical Engineering, Tel Aviv University, Tel Aviv, Israel; 2 Sagol School of Neuroscience, Tel Aviv University, Tel Aviv, Israel; 3 The Center for Nanoscience and Nanotechnology, Tel Aviv University, Tel Aviv, Israel; 4 Department of Microsystems Engineering (IMTEK), University of Freiburg, Freiburg, Germany; 5 Center BrainLinks-BrainTools, University of Freiburg, Freiburg, Germany; 6 Division of Nursing and Medical Technology, Luleå University of Technology, Lulea, Sweden; 7 Department of Neurology, The Chaim Sheba Medical Center, Tel Hashomer, Israel; 8 Department of Neurology and Neurosurgery, Sackler Faculty of Medicine and Sagol School of Neuroscience, Tel Aviv University, Israel; 9 Department of Neuroanatomy, Institute of Anatomy and Cell Biology, Faculty of Medicine, University of Freiburg, Freiburg, Germany; 10 Center for Basics in Neuromodulation (NeuroModulBasics), Faculty of Medicine, University of Freiburg, Freiburg, Germany

**Keywords:** Thrombin, PAR1, Blood brain barrier, Neurovascular unit, Organ-on-chips, Sensors, Human relevant in vitro models

## Abstract

Blood coagulation factors can enter the brain under pathological conditions that affect the blood–brain interface. Besides their contribution to pathological brain states, such as neural hyperexcitability, neurodegeneration, and scar formation, coagulation factors have been linked to several physiological brain functions. It is for example well established that the coagulation factor thrombin modulates synaptic plasticity; it affects neural excitability and induces epileptic seizures via activation of protease-activated receptors in the brain. However, major limitations of current experimental and clinical approaches have prevented us from obtaining a profound mechanistic understanding of “neuro-coagulation” in health and disease. Here, we present how novel human relevant models, i.e., Organ-on-Chips equipped with advanced sensors, can help overcoming some of the limitations in the field, thus providing a perspective toward a better understanding of neuro-coagulation in brain homeostasis.

## Introduction

A defining feature of complex biological systems is the intricate interplay between spatially and temporally segregated signaling pathways that are based on positive and negative feedback mechanisms. Over the past century, major advances were made in specific biological systems and organ systems by identifying important signaling pathways relevant for various biological functions. In several cases, new targets for therapeutic interventions could be identified and drugs were developed for the treatment of diseases, associated with alterations in the respective signaling pathways and associated organ functions. It has been also proposed that some of the relevant signaling molecules are exclusively expressed in the respective organ systems. Accordingly, generations of scientists and medical doctors have been educated with a molecular understanding of signaling pathways that have been assigned to specific organs and their functions (Berg et al. 2002). The *blood coagulation cascade* (Macfarlane 1964; Davie and Ratnoff 1964) is a paradigmatic example for such “branding” of enzymes, substrates, and signaling pathways, as reflected also by nomenclature.

Not surprising, therefore, that for a long time, the relevance of coagulation factors has not been considered in the field of neuroscience, at least as regard to their role in defining physiological brain functions such as neural excitability and synaptic plasticity. Certainly, the identification of the blood–brain interface (BBI; Hajal et al. 2021; Schaeffer and Iadecola 2021; Segarra et al. 2021), which limits and controls the exchanges of molecules and cells between the body and the central nervous system, has contributed to stalling scientific advances in this field of research; coagulation factors such as Factor Xa or Factor IIa, i.e., thrombin, for example, do not enter the brain under physiological conditions. However, recent discoveries have demonstrated the expression of coagulation factors and protease activated receptors (PARs) in the brain (reviewed in Ben Shimon et al. (2015)). Since pathological conditions are often accompanied by alterations in BBI functions, and consequently an increase in blood components in the brain, these findings have led to a series of exciting new interdisciplinary studies with considerable clinical relevance.

Accordingly, several brain diseases have been associated with changes in the expression of PARs in the brain, such as Parkinson’s disease and Alzheimer’s disease (Grossmann 2021; Iannucci et al. 2020). Furthermore, the physiological roles of thrombin and PAR-mediated signaling pathways in neural excitability and synaptic plasticity have been studied, and major findings on the biological significance of coagulation factors in brain physiology and pathology have been systematically reviewed elsewhere (e.g., Ben Shimon et al. 2015; De Luca et al. 2017; Shlobin et al. 2021). Considering the limitations of current experimental approaches, here we discuss how the development of novel human relevant technologies, i.e., Organ-on-Chips equipped with next generation sensors, could help in advancing the field toward a better understanding of the role of neuro-coagulation in brain homeostasis at the interface between health and disease.

## Vicious cycles at the neurovascular unit and the role of thrombin/PAR1 in synaptic plasticity

**Fig. 1:**
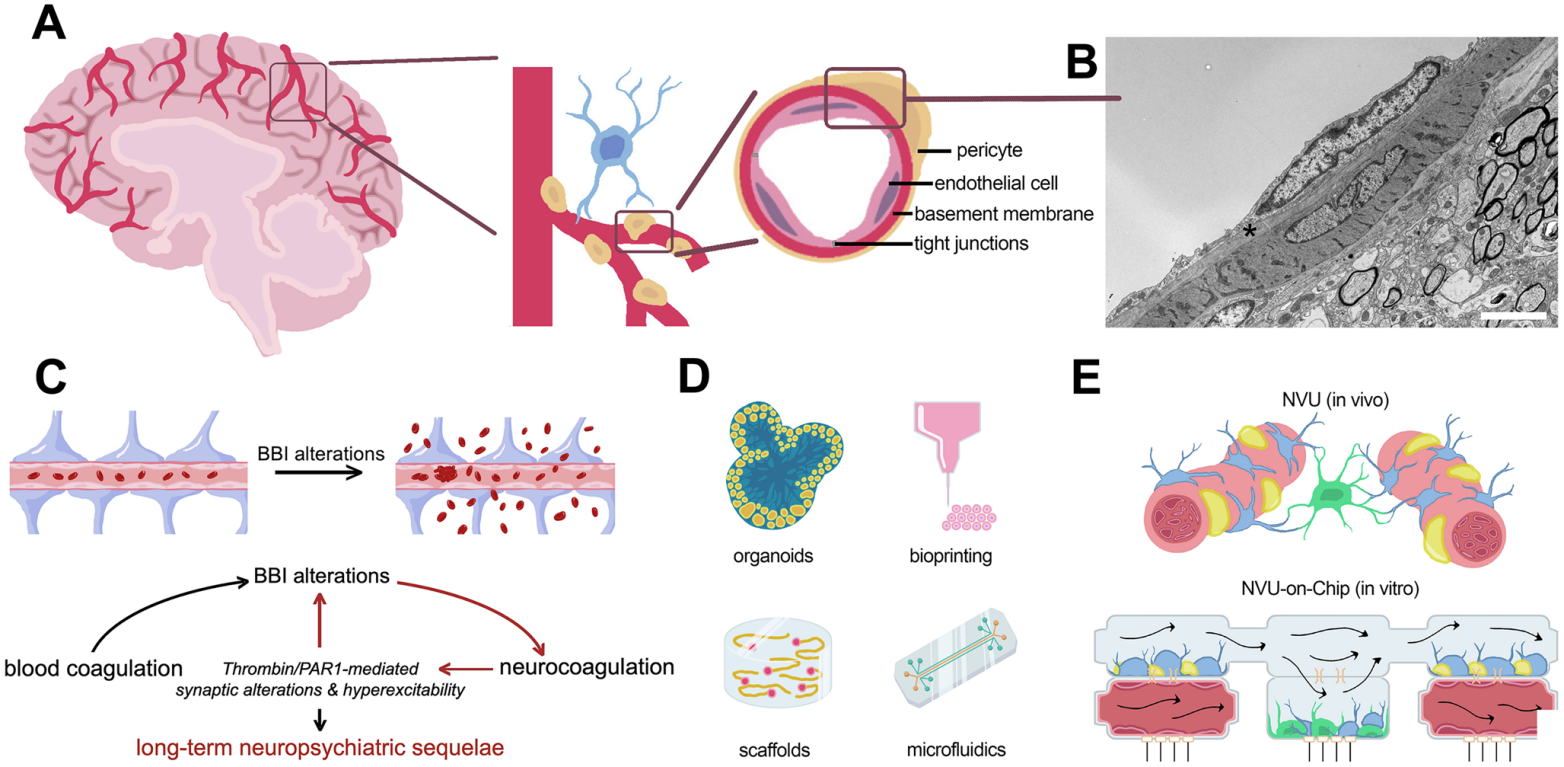
Neuro-coagulation and the neurovascular unit (NVU). **A** Schematic illustrating cellular components of the blood brain interface. Astrocyte in blue. **B** Electron micrograph of a vessel in the brain. Note the basement membrane between endothelial cell and pericyte (asterisk). Scale bar = 2.5 µm. **C** Vicious cycle at the neurovascular unit (for details see text). **D** Illustrations of advanced human in vitro models. **E** The use of Organs-on-a-Chip (OoC) for studying the NVU. Equipped with advanced sensors, OoCs provide a technology-based approach toward better understanding of neuro-coagulation in brain homeostasis

The brain is a multi-scale system with several layers of complexity at the molecular, cellular, and network level. One of the most fundamental units of the brain is the neurovascular unit (NVU), which comprises major functional interactions of the brain vasculature and parenchyma (Schaeffer and Iadecola 2021). The NVU is a densely packed multi-cellular biophysical system formed by the following main populations of cells: the brain microvascular endothelium, the perivascular space composed of pericytes and astrocytes, that create the BBI, and the adjacent brain parenchyma containing complex glial and neuronal networks (Fig. 1A, B). Clinical and experimental evidence suggests that alterations in BBI function, which controls the transport of chemicals, cells, and nutrients from the blood to the brain and vice versa, may represent a major pathophysiological hallmark of brain disease. Indeed, there is an indisputable correlation between BBI alterations and brain diseases (Abrahamson and Ikonomovic 2020; Chodobski et al. 2011).

Conditions leading to BBI breakdown impair BBI selectivity and allow the entrance of blood-derived molecules and cells into the brain. Work from recent years suggests that alterations in BBI and subsequent cortical exposure to blood constituents may initiate, promote, and/or sustain long-lasting structural, functional, and molecular changes in the neural tissue (Petersen et al. 2018; Shlosberg et al. 2010). Since molecular players of the coagulation cascade are expressed also in the brain (c.f., Ben Shimon et al. 2015)), in turn, “brain born” coagulation factors (and inflammatory cytokines) related to pathological changes in network activity may trigger BBI alterations. Additional coagulation factors entering the brain from the vascular system may sustain and exacerbate the physiological effects of neuro-coagulation under these conditions, leading to a detrimental vicious cycle at the NVU (Fig. 1C).

Experimental evidence for such a vicious cycle at the NVU comes from pharmacological studies in acute hippocampal slices that have identified a role of thrombin and PAR1-signaling in neural excitability (Maggio et al. 2013a). PARs are G-coupled receptors that are activated by a site-specific proteolytic cleavage of their N-terminal extracellular domain, which uncovers a tethered ligand. The serine protease thrombin is one of the major activators of PAR1, PAR3, and PAR4; PAR2 represents a class of trypsin/tryptase activated receptors (Gingrich and Traynelis 2000). In the brain, high levels of PAR1 are detected in the cortex, hippocampus, and striatum (Junge et al. 2004). Indeed, thrombin-mediated activation of PAR1 enhances NMDA-R currents; it affects synaptic plasticity and increases neural excitability (Ben Shimon et al. 2015). Experimental evidence has been provided showing that pharmacological inhibition of thrombin and PAR1 assert positive effects; e.g., it restores the ability of neurons to express synaptic plasticity under conditions of increased brain thrombin levels (Becker et al. 2014; Maggio et al. 2008). Moreover, it was shown that recovery from trauma-induced amnesia correlates with a normalization of thrombin activity in the mouse brain (Itzekson et al. 2014).

Interestingly, dose-dependent effects of thrombin/PAR1 activation were reported. Specifically, it was shown that high concentrations of thrombin saturate the ability of neurons to express plasticity via PAR1-activation (Becker et al. 2014; Maggio et al. 2008), while at low concentration, thrombin enhances plasticity via activated protein C and the Endothelial Protein C Receptor (Maggio et al. 2013b, 2014, 2008). Apparently, under conditions of BBI alterations, an increase in brain thrombin levels will override the plasticity promoting effects of low thrombin/PAR1 activation, thus causing alterations in complex brain function.

## Role of thrombin/PAR1 in hyperexcitability and status epilepticus

Hyperexcitability and seizures have been associated with a rapid and robust increase in BBI permeability (Ruber et al. 2018; Swissa et al. 2019). Therefore, it has been hypothesized that the brain may be exposed to blood components that could promote further excitability, neuroinflammation, and ultimately maladaptive network modifications and epileptogenesis (de Vries et al. 2012; Seiffert et al. 2004). Among the candidates, thrombin and the PAR1 pathway seem to play a pivotal role in these mechanisms.

Indeed, it was shown that application of thrombin on hippocampal slices induces hyperexcitability through a PAR1-NMDA receptor mediated mechanism (Maggio et al. 2008). In the context of post-traumatic brain injury (TBI), which affects BBI function, thrombin was found to lower the seizure threshold in an animal model of TBI possibly contributing to the mechanism of post-traumatic seizures (Altman et al. 2019; Ben Shimon et al. 2019). In an animal model of organophosphate-induced status epilepticus, a high level of thrombin activity in the hippocampus was detected; furthermore, organophosphate-induced hyperexcitability in the hippocampus was markedly reduced by a specific PAR1 antagonist (SCH79797; Golderman et al. 2019). Finally, systemic thrombin inhibition ameliorated seizures in a mouse model of status epilepticus (Lenz et al. 2019). Altogether, these studies have put forward the intriguing hypothesis that possibly novel oral anticoagulants targeting thrombin and its PAR1 pathway may assert antiepileptic effects in vivo. It is clear that a better understanding of neuro-coagulation and the underlying cellular and molecular mechanisms of its impact on complex NVU functions may help in devising new strategies for predicting, preventing, and treating brain diseases associated with alteration in NVU/BBI function.

## Major limitations of current experimental and clinical models

Current experimental in vivo models are limited as it is experimentally challenging to monitor the spatial and temporal dynamics of the NVU and its distinct components in situ. Specifically, it is not trivial to readily distinguish structural, functional, and metabolic interactions among the neural cells embedded in the brain parenchyma, the perivasculature, and the microvasculature in healthy tissue or pathological conditions. Animal models also show significant inter-species differences which include differences in efflux transporters, tight junctions, and cell–cell signaling (Benigni 2016). Due to ethical and technical constraints, invasive in vivo experiments are impossible in healthy subjects and patients. The possibilities to measure the BBI function and the effects of coagulation factors in humans are very limited. Clinicians rely on information that can be collected mainly non-invasively (e.g., computed tomography, magnetic resonance imaging, near infrared spectroscopy), or on biomarkers that can reveal a compromised BBI only indirectly (Raja et al. 2018).

While current in vitro models are more accessible, as simplified systems, they also have several major limitations with regard to studying BBI and NVU functions. For example, dissociated primary neuronal cultures and cell cultures derived from human-inducible pluripotent stem cells (hiPSCs) lack important cellular and three-dimensional (3D) anatomical complexity. They hardly create or maintain 3D vascular structures, and adjacent pericytes, astrocytes, and basal membranes do not form. Organotypic tissue cultures demonstrate some of these cellular features. However, they are blood-free preparations. Vasculature either does not form properly as seen in the case of iPSC-derived organoids, or it degenerates in the absence of physiological blood flow in cultured brain slices (Chip et al. 2013). Properly connecting vasculature and capillaries of explanted brain tissue to microfluidic devices, and carefully controlling intravascular pressure, perfusion, and shear forces, which are crucial for proper development and maintenance of the vasculature and neural tissue, is technically demanding and has not yet been sufficiently established. This emphasizes that the current methods and models used to assess NVU function in health and disease (both in vivo and in vitro) are insufficient or not suitable for translational studies (c.f., Maoz 2021).

## Novel human relevant technologies for studying neuro-coagulation on a chip

In recent years, a major leap forward has been made in advancing in vitro human relevant brain models (Nikolakopoulou et al. 2020). These models include the use of 3D-printing technologies, hiPSC-derived organoids, advanced microfabrication and nanofabrication, and tissue engineering (Fig. 1D). A promising new technology for culturing cells and tissue on dedicated microfluidic chips is known as Organ-on-a-Chip (OoC) and/or micro-physiological systems (MPS; Fig. 1D, E). This new concept allows to recapitulate in vivo physiological conditions in a unique micro environment which would not be possible to achieve using conventional culturing models (e.g., flow/shear, mechanical cues, physiochemical micro environment, and the ability to link several OoC to create mini-human-physiological-systems-on-a-Chip allowing organ-organ interactions; Novak et al. 2020).

One of the major strengths of this technology is the ability to decouple complex human physiological systems such as the NVU while keeping it metabolically coupled. Indeed, a human NVU-on-a-Chip was recently developed and used to identify the metabolic coupling between the brain microvasculature and neurons (Maoz et al. 2018) (Fig. 1E). Another advantage of the OoC system is the ability to induce flow and shear, which is a fundamental property of the vascular system that is lacking in a conventional in vitro system. Example for such use was provided by Nemocovsky Amar et al. in 2019, who created an ischemia–reperfusion injury microfluidic chip with blood coagulation (Nemcovsky Amar et al. 2019). Other OoC systems demonstrate the ability to perfuse human blood in the chip for studying changes in the BBI permeability (Vatine et al. 2019).

In this context, it is important to consider the implementation of tools and sensors that allow for in situ monitoring of relevant functional parameters (e.g., changes in the permeability of the BBI, metabolite concentrations, and structural and functional plasticity of neurons and glia). While the development and implementation of such sensors is not trivial, recent developments in sensor fabrication and microfabrication and nanofabrication allow integration of sophisticated sensors in OoC (Zhu et al. 2021).

## Advanced sensors for studying neuro-coagulation

Key to monitoring and probing neuro-coagulation and other complex NVU mechanisms are accurate real-time functional recordings over extended periods of time. Sensors ideally should allow for nearly unrestricted sampling of the neural activity across the larger networks as well as high spatial resolution recordings within small subsets of the population. Furthermore, the electrophysiological data should be complemented with information on the micro environment, such as conductivity/permeability of the BBI, local pH variations, the presence of reactive oxygen species (ROS), and specific molecules (e.g., Factor Xa or Factor IIa), all highly relevant parameters in neuro-coagulation and inflammation.

Recent advances in microfabrication and nanofabrication have enabled the development of sensors with high sensitivity, selectivity, and robustness while keeping dimensions small. These advanced sensors preferably should access all planes of the tissue volume or even be embedded within the tissue itself. Furthermore, it is imperative that the implementation of these distributed and integrated sensors does not come at the cost of perfusion or optical access (e.g., for structural and functional imaging), meaning the architecture must be maximally open.

Long-term stable high-resolution sampling and artificial excitation of neuronal signals are now available by the use of flexible sensor microprobes based on polymeric substrates such as polyimide (Chung et al. 2019; Luan et al. 2017). The main advantage of flexible microtechnology is that it allows for excellent spatial resolution to be combined with light-weight devices which, due to their mechanical flexibility, easily conform to soft three-dimensional objects such as the brain surface in vivo, or a tissue culture in vitro (Vomero et al. 2020). Although flexible neural probes have revolutionized in vivo implantable neurotechnology, current systems for in vitro recordings have not yet drawn full advantage of this development. Thus, this field must capitalize on the rapid progress within neurotechnology to advance the electrophysiology and biosensing function of OoC technology platforms. Recent work shows great promise, e.g., a 3D flexible MEA-platform, which was able to form a three-dimensional interface to a brain model including both astrocytes and neurons in a hydrogel matrix (Soscia et al. 2020). McDonald et al. showed how a flexible mesh-like MEA-array can be used to interface brain organoids (McDonald et al. 2021).

As medical science becomes more interdisciplinary, the development and integration of novel advanced sensors into innovative translational OoC-platforms will allow us to identify and probe complex cell–cell interactions at the NVU. We are confident that this technology-based approach will shed new important light on the mechanism according to which neuro-coagulation affects complex brain function in health and diseases, such as traumatic brain injury, epilepsy, and neurodegeneration.

## References

[CR1] Abrahamson EE, Ikonomovic MD (2020). Brain injury-induced dysfunction of the blood brain barrier as a risk for dementia. Exp Neurol.

[CR2] Altman K, Shavit-Stein E, Maggio N (2019). Post stroke seizures and epilepsy: from proteases to maladaptive plasticity. Front Cell Neurosci.

[CR3] Becker D, Ikenberg B, Schiener S, Maggio N, Vlachos A (2014). NMDA-receptor inhibition restores Protease-Activated Receptor 1 (PAR1) mediated alterations in homeostatic synaptic plasticity of denervated mouse dentate granule cells. Neuropharmacology.

[CR4] Ben Shimon M, Lenz M, Ikenberg B, Becker D, Shavit Stein E, Chapman J, Tanne D, Pick CG, Blatt I, Neufeld M (2015). Thrombin regulation of synaptic transmission and plasticity: implications for health and disease. Front Cell Neurosci.

[CR5] Ben Shimon M, Shavit-Stein E, Altman K, Pick CG, Maggio N (2019). Thrombin as key mediator of seizure development following traumatic brain injury. Front Pharmacol.

[CR6] Benigni R (2016). Predictive toxicology today: the transition from biological knowledge to practicable models. Expert Opin Drug Metab Toxicol.

[CR7] Berg JM, Tymoczko JL, Stryer L, Stryer L (2002). Biochemistry.

[CR8] Chip S, Nitsch C, Wellmann S, Kapfhammer JP (2013). Subfield-specific neurovascular remodeling in the entorhino-hippocampal-organotypic slice culture as a response to oxygen-glucose deprivation and excitotoxic cell death. J Cereb Blood Flow Metab.

[CR9] Chodobski A, Zink BJ, Szmydynger-Chodobska J (2011). Blood-brain barrier pathophysiology in traumatic brain injury. Transl Stroke Res.

[CR10] Chung JE, Joo HR, Fan JL, Liu DF, Barnett AH, Chen S, Geaghan-Breiner C, Karlsson MP, Karlsson M, Lee KY et al (2019) High-density, long-lasting, and multi-region electrophysiological recordings using polymer electrode arrays. Neuron 101:21–31 10.1016/j.neuron.2018.11.002 PMC6326834 30502044

[CR11] Davie EW, Ratnoff OD (1964). Waterfall sequence for intrinsic blood clotting. Science.

[CR12] De Luca C, Virtuoso A, Maggio N, Papa M (2017) Neuro-coagulopathy: blood coagulation factors in central nervous system diseases. Int J Mol Sci 18 10.3390/ijms18102128 PMC5666810 29023416

[CR13] de Vries HE, Kooij G, Frenkel D, Georgopoulos S, Monsonego A, Janigro D (2012). Inflammatory events at blood-brain barrier in neuroinflammatory and neurodegenerative disorders: implications for clinical disease. Epilepsia.

[CR14] Gingrich MB, Traynelis SF (2000). Serine proteases and brain damage — is there a link?. Trends Neurosci.

[CR15] Golderman V, Shavit-Stein E, Gera O, Chapman J, Eisenkraft A, Maggio N (2019). Thrombin and the protease-activated receptor-1 in organophosphate-induced status epilepticus. J Mol Neurosci.

[CR16] Grossmann K (2021) Alzheimer’s disease-rationales for potential treatment with the thrombin inhibitor dabigatran. Int J Mol Sci 22 10.3390/ijms22094805 PMC8125318 33946588

[CR17] Hajal C, Le Roi B, Kamm RD, Maoz BM (2021). Biology and models of the blood-brain barrier. Annu Rev Biomed Eng.

[CR18] Iannucci J, Renehan W, Grammas P (2020). Thrombin, a mediator of coagulation, inflammation, and neurotoxicity at the neurovascular interface: implications for Alzheimer’s disease. Front Neurosci.

[CR19] Itzekson Z, Maggio N, Milman A, Shavit E, Pick CG, Chapman J (2014). Reversal of trauma-induced amnesia in mice by a thrombin receptor antagonist. J Mol Neurosci.

[CR20] Junge CE, Lee CJ, Hubbard KB, Zhang Z, Olson JJ, Hepler JR, Brat DJ, Traynelis SF (2004). Protease-activated receptor-1 in human brain: localization and functional expression in astrocytes. Exp Neurol.

[CR21] Lenz M, Shimon MB, Benninger F, Neufeld MY, Shavit-Stein E, Vlachos A, Maggio N (2019). Systemic thrombin inhibition ameliorates seizures in a mouse model of pilocarpine-induced status epilepticus. J Mol Med (berl).

[CR22] Luan L, Wei X, Zhao Z, Siegel JJ, Potnis O, Tuppen CA, Lin S, Kazmi S, Fowler RA, Holloway S et al (2017) Ultraflexible nanoelectronic probes form reliable, glial scar-free neural integration. Sci Adv 3:e1601966 10.1126/sciadv.1601966 PMC5310823 28246640

[CR23] Macfarlane RG (1964). An enzyme cascade in the blood clotting mechanism, and its function as a ciochemical amplifier. Nature.

[CR24] Maoz BM (2021) Brain-on-a-Chip: Charaterizing the next generation of advanced in vitro platforms for modeling the central nervous system. APL Bioeng 5:030902 10.1063/5.0055812 PMC8325567 34368601

[CR25] McDonald M, Sebinger D, Brauns L, Gonzalez-Cano L, Menuchin-Lasowski Y, Mierzejewski M, Psathaki O-E, Stumpf A, Wickham J, Rauen T, Schöler H, Jones PD (2021) A mesh microelectrode array for non-invasive electrophysiology within neural organoids, bioRxiv 2020−09 10.1016/j.bios.2023.115223 36931193

[CR26] Maggio N, Blatt I, Vlachos A, Tanne D, Chapman J, Segal M (2013). Treating seizures and epilepsy with anticoagulants?. Front Cell Neurosci.

[CR27] Maggio N, Itsekson Z, Dominissini D, Blatt I, Amariglio N, Rechavi G, Tanne D, Chapman J (2013). Thrombin regulation of synaptic plasticity: implications for physiology and pathology. Exp Neurol.

[CR28] Maggio N, Itsekson Z, Ikenberg B, Strehl A, Vlachos A, Blatt I, Tanne D, Chapman J (2014). The anticoagulant activated protein C (aPC) promotes metaplasticity in the hippocampus through an EPCR-PAR1-S1P1 receptors dependent mechanism. Hippocampus.

[CR29] Maggio N, Shavit E, Chapman J, Segal M (2008). Thrombin induces long-term potentiation of reactivity to afferent stimulation and facilitates epileptic seizures in rat hippocampal slices: toward understanding the functional consequences of cerebrovascular insults. J Neurosci.

[CR30] Maoz BM, Herland A, FitzGerald EA, Grevesse T, Vidoudez C, Pacheco AR, Sheehy SP, Park TE, Dauth S, Mannix R (2018). A linked organ-on-chip model of the human neurovascular unit reveals the metabolic coupling of endothelial and neuronal cells. Nat Biotechnol.

[CR31] Nemcovsky Amar D, Epshtein M, Korin N (2019) Endothelial Cell Activation in an Embolic Ischemia-Reperfusion Injury Microfluidic Model. Micromachines (Basel) 10 10.3390/mi10120857 PMC6952880 31817733

[CR32] Nikolakopoulou P, Rauti R, Voulgaris D, Shlomy I, Maoz BM, Herland A (2020). Recent progress in translational engineered in vitro models of the central nervous system. Brain.

[CR33] Novak R, Ingram M, Marquez S, Das D, Delahanty A, Herland A, Maoz BM, Jeanty SSF, Somayaji MR, Burt M (2020). Robotic fluidic coupling and interrogation of multiple vascularized organ chips. Nat Biomed Eng.

[CR34] Petersen MA, Ryu JK, Akassoglou K (2018). Fibrinogen in neurological diseases: mechanisms, imaging and therapeutics. Nat Rev Neurosci.

[CR35] Raja R, Rosenberg GA, Caprihan A (2018). MRI measurements of blood-brain barrier function in dementia: A review of recent studies. Neuropharmacology.

[CR36] Ruber T, David B, Elger CE (2018). MRI in epilepsy: clinical standard and evolution. Curr Opin Neurol.

[CR37] Schaeffer S, Iadecola C (2021) Revisiting the neurovascular unit. Nat Neurosci 10.1038/s41593-021-00904-7 PMC9462551 34354283

[CR38] Segarra M, Aburto MR, Acker-Palmer A (2021). Blood-brain barrier dynamics to maintain brain homeostasis. Trends Neurosci.

[CR39] Seiffert E, Dreier JP, Ivens S, Bechmann I, Tomkins O, Heinemann U, Friedman A (2004). Lasting blood-brain barrier disruption induces epileptic focus in the rat somatosensory cortex. J Neurosci.

[CR40] Shlobin NA, Har-Even M, Itsekson-Hayosh Z, Harnof S, Pick CG (2021) Role of Thrombin in Central Nervous System Injury and Disease. Biomolecules 11 10.3390/biom11040562 PMC8070021 33921354

[CR41] Shlosberg D, Benifla M, Kaufer D, Friedman A (2010). Blood-brain barrier breakdown as a therapeutic target in traumatic brain injury. Nat Rev Neurol.

[CR42] Soscia DA, Lam D, Tooker AC, Enright HA, Triplett M, Karande P, Peters SKG, Sales AP, Wheeler EK, Fischer NO (2020). A flexible 3-dimensional microelectrode array for in vitro brain models. Lab Chip.

[CR43] Swissa E, Serlin Y, Vazana U, Prager O, Friedman A (2019). Blood-brain barrier dysfunction in status epileptics: mechanisms and role in epileptogenesis. Epilepsy Behav.

[CR44] Vomero M, Porto-Cruz M, Zucchini E, Ciarpella F, Delfino E, Carli S, Boehler C, Asplund M, Ricci D, Fadiga L, Stieglitz T (2020) Conformable polyimide-based μECoGs: bringing the electrodes closer to the signal source. Biomaterials 255:120178 10.1016/j.biomaterials.2020.120178 32569863

[CR45] Vatine GD, Barrile R, Workman MJ, Sances S, Barriga BK, Rahnama M, Barthakur S, Kasendra M, Lucchesi C, Kerns J et al (2019) Human iPSC-derived blood-brain barrier chips enable disease modeling and personalized medicine applications. Cell Stem Cell 24:995–1005 10.1016/j.stem.2019.05.011 31173718

[CR46] Zhu Y, Mandal K, Hernandez AL, Kawakita S, Huang W, Bandaru P, Ahadian S, Kim H, Jucaud V, Dokmeci MR, Khademhosseini A (2021) State of the art in integrated biosensors for organ-on-chip applications. Curr Opin Biomed Eng 19:100309 10.1016/j.cobme.2021.100309 PMC10193909 37206309

